# A Rare Case of Pulmonary Metastasis From Intracranial Grade 2 Meningioma

**DOI:** 10.7759/cureus.103080

**Published:** 2026-02-06

**Authors:** Vikas Kumar Pandey, Abdulmajeed Dayyat, Marc Del Bigio, Saranya Kakumanu

**Affiliations:** 1 Radiation Oncology, University of Manitoba, Winnipeg, CAN; 2 Pathology, University of Manitoba, Winnipeg, CAN; 3 Radiation Oncology, CancerCare Manitoba, Winnipeg, CAN

**Keywords:** atypical meningioma, brain invasion, fractionated stereotactic radiosurgery, meningioma, pulmonary metastases

## Abstract

Meningiomas are the most common benign central nervous system tumors in adults. Meningiomas are typically categorized into three grades that reflect their aggressiveness, recurrence risk, and clinical prognosis. Although these tumors have low metastatic potential, the prognosis is significantly worse when metastases occur. Due to the scarcity of available literature on metastasis, the exact cause and treatment of metastatic disease are poorly understood. We present a rare case of a 62-year-old male with atypical meningioma with early pulmonary metastasis shortly after standard-of-care treatment, highlighting the importance of vigilant imaging surveillance in high-risk disease.

## Introduction

Meningiomas account for approximately 30% of all primary intracranial tumors in adults and are predominantly benign (WHO Grade 1) [1]. However, WHO Grade 2 (atypical) and Grade 3 (malignant/anaplastic) variants demonstrate higher recurrence rates and a greater potential for aggressive clinical behavior [2]. Extracranial metastasis is extremely rare, with a reported incidence of <1% [3]. The lungs, bones, and lymph nodes are the most common metastatic sites, and the presence of metastases significantly reduces five-year overall survival [4].

Diagnostic criteria for meningiomas continue to evolve. In the 2016 WHO classification, brain invasion was added as a defining criterion for Grade 2 meningiomas [5], and in 2021, a molecular-based classification system further refined diagnostic categories [6].

Here, we describe a rare case of early pulmonary metastasis from a WHO Grade 2 meningioma shortly after standard therapy. This case report highlights the unusual recurrence of a biopsy-confirmed lung metastasis from a WHO Grade 2 meningioma and opens discussions on surveillance in high-risk meningioma patients. High-risk meningiomas are commonly defined as newly diagnosed or recurrent WHO Grade 3 tumors (any extent of resection), recurrent WHO Grade 2 tumors (any extent of resection), or newly diagnosed WHO Grade 2 tumors following subtotal resection.

## Case presentation

Case description

A 62-year-old male with multiple chronic comorbidities, including diabetes mellitus with recurrent episodes of hyperglycemia and a history of left lower limb amputation, presented in September 2024 with loss of consciousness, confusion, and seizure. On physical examination, his Eastern Cooperative Oncology Group (ECOG) performance status was 2. No focal neurological deficits were noted.

**Figure 1:**
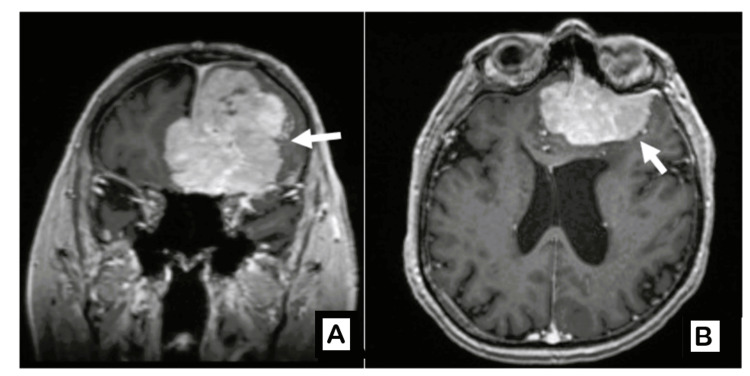
Preoperative contrast-enhanced T1-weighted MRI in coronal (A) and axial (B) views showing a large anterior cranial fossa meningioma.

MRI of the brain revealed a large anterior cranial fossa/frontal meningioma measuring 6.3 × 4.9 × 6.8 cm, associated with significant mass effect (Figure 1). The patient underwent craniotomy under neuronavigation with gross total tumor resection.

**Figure 2:**
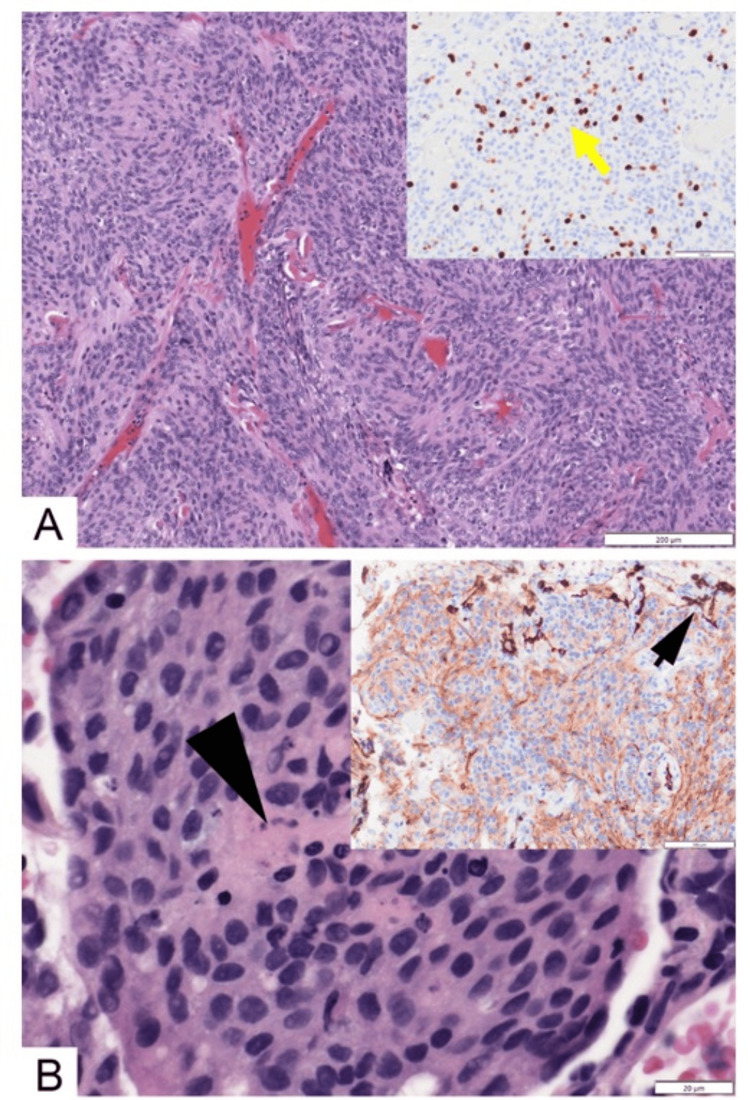
(A) Photomicrograph showing intracranial meningioma (hematoxylin and eosin stain; original magnification 100x). The inset shows Ki67 immunostain with high proliferation activity (yellow arrow, brown diaminobenzidine with blue hematoxylin counterstain; original magnification 100×). (B) Photomicrograph showing meningioma metastatic to the lung. The center of the whorl-like complex is necrotic (black arrowhead) (hematoxylin and eosin stain; original magnification 600×). The inset shows epithelial membrane antigen (EMA) immunostain. The dark-colored cells (black arrow) are resident pneumocytes, and the moderately brown-colored cells are the meningothelial cells (brown diaminobenzidine with blue hematoxylin counterstain; original magnification 200×).

Postoperative histopathological analysis demonstrated a WHO Grade 2 meningioma with a high proliferative index and evidence of brain invasion (Figure 2A). Postoperatively, the patient recovered well without any neurological deficits, except for intermittent urinary incontinence. The first postoperative MRI, performed on the day following surgery, showed no evidence of residual or recurrent disease.

**Figure 3:**
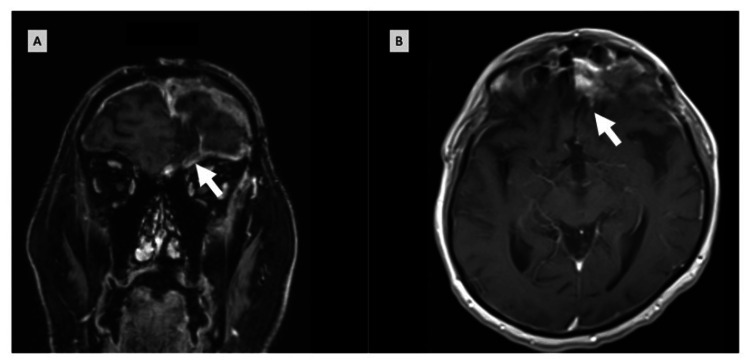
MRI five months after surgery, showing progressive enhancement in coronal (A) and axial (B) views.

Five months following surgery, surveillance MRI revealed progressive enhancement within the right anterior cranial fossa along the cribriform plate, concerning for recurrent disease (Figure 3). He subsequently received salvage fractionated stereotactic radiosurgery (FSRS) to the residual lesion in the brain at a dose of 30 Gy in 5 fractions.

Approximately one month after completion of FSRS, the patient presented with one week of new-onset, sudden, localized chest pain of a non-pleuritic nature, occurring intermittently, along with mild dyspnea on exertion. There was no history of fever, cough, hemoptysis, palpitations, or weight loss.

**Figure 4:**
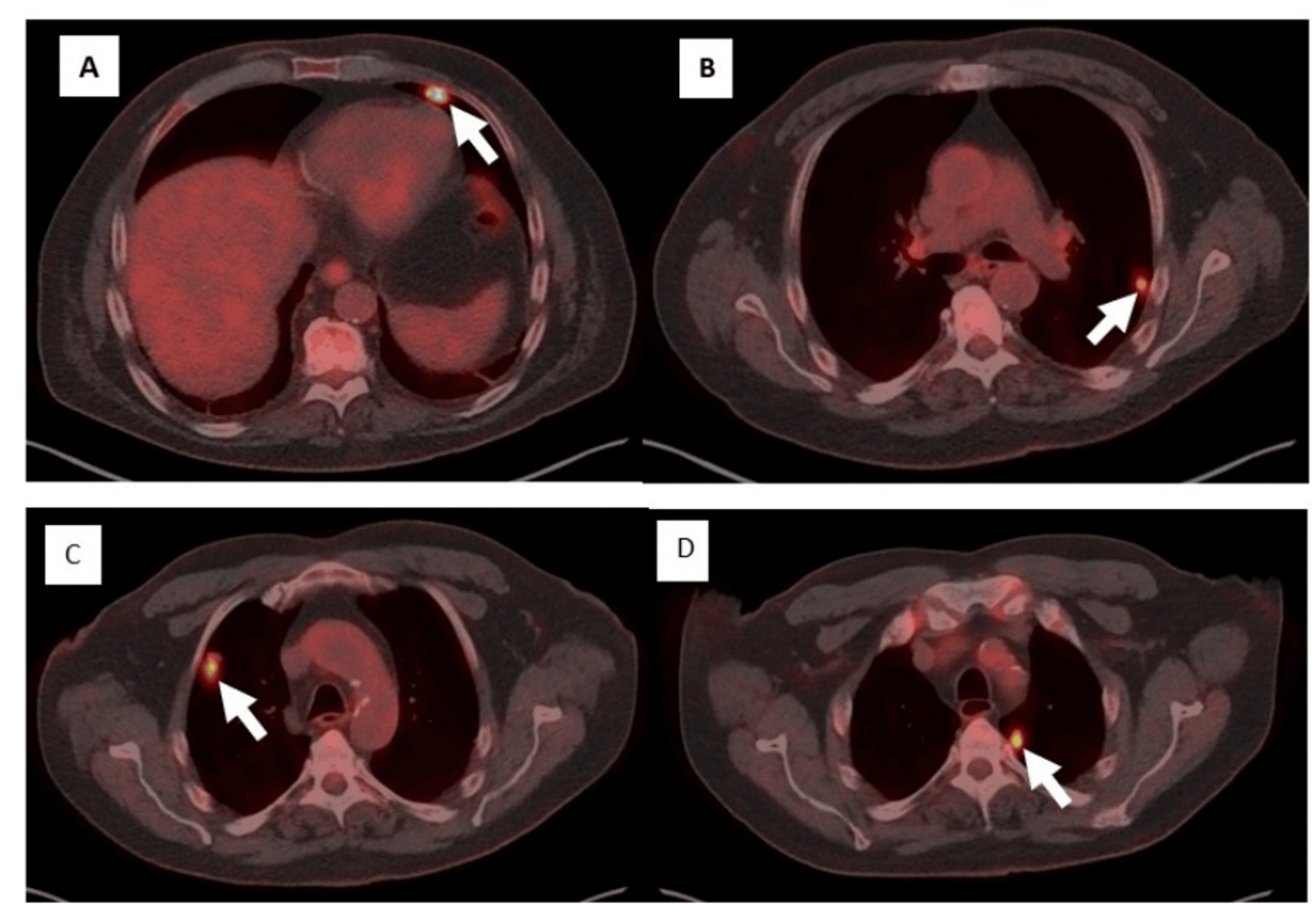
Whole-body PET scan demonstrating multiple bilateral pulmonary nodules consistent with metastatic meningioma (A–D). PET: positron emission tomography

On examination, his ECOG performance status remained at 2. Respiratory examination revealed occasional crepitations in the left lower lobe, with otherwise normal vesicular breath sounds. A chest CT scan demonstrated an incidental finding of bilateral pulmonary nodules. A whole-body positron emission tomography (PET) revealed multiple hypermetabolic bilateral pulmonary nodules, highly suspicious for metastatic disease (Figure 4).

Given the suspicious lung lesions, a right upper lobe lung biopsy was performed, which confirmed metastatic meningioma (Figure 2B). His symptoms resolved with conservative management. In view of the absence of established systemic therapy options and the patient being asymptomatic, a decision was made for close observation with repeat imaging after three months.

On continued follow-up after three months, the patient demonstrated progression of pulmonary metastases with stable intracranial disease. The option of stereotactic radiotherapy for the lung lesions versus continued observation was discussed with the patient. As he was asymptomatic, he elected to proceed with observation. He is currently on a three-month follow-up with serial imaging of the brain and chest.

## Discussion

Since the first reported case of meningioma in 1886, extracranial metastases have remained exceedingly rare [2,7]. Their underlying mechanisms remain unclear due to limited data, with contemporary estimates suggesting a metastatic prevalence of approximately 0.18% [3]. The most common sites of metastasis are the lungs, followed by the bones and the liver [8]. Given the rarity of extracranial dissemination, treatment is largely individualized, with limited evidence supporting systemic therapy such as hydroxyurea, somatostatin analogues, or targeted agents.

Tumor grade (Grade 3 and less likely Grade 2) remains the strongest predictor of metastatic potential [4]. Other factors include venous sinus invasion and open cranial surgery, which may facilitate hematogenous spread [9]. Garzon-Muvdi et al. suggested that cranioplasty for these patients may serve as a focal point for scalp invasion and recommended the use of nonporous materials where appropriate [10].

In the present case, the presence of brain invasion in a Grade 2 tumor extending to the superior sagittal sinus likely contributed to metastatic dissemination. The rapid appearance of pulmonary metastases shortly after radiosurgery emphasizes the need for close surveillance in high-risk meningiomas. Recent studies have emphasized the role of molecular profiling in understanding the metastatic potential of meningiomas. Alterations such as telomerase reverse transcriptase (TERT) promoter mutations and cyclin-dependent kinase inhibitor 2A/B (CDKN2A/B) deletions have been associated with aggressive tumor biology and poorer prognosis, even in cases histologically graded as atypical (Grade 2). These genetic changes may offer predictive insights into early recurrence or distant metastasis, thereby guiding individualized surveillance and therapeutic strategies [11,12]. In our case, CDKN2A/B or TERT testing was not done.

Technical innovations such as 68Ga-DOTATATE PET imaging may enhance the identification of meningioma metastases. Utilizing the well-documented association between meningiomas and somatostatin receptor 2 (SSTR2) expression, 68Ga-DOTATATE employs an SSTR2 mimic to differentiate postoperative and post-radiation alterations from meningioma tumor tissue [13]. It helps identify metastatic lesions by highlighting biologically active meningioma cells through their SSTR2 expression, allowing detection of even small or atypical lesions. In 2015, Rachinger et al. demonstrated that 68Ga-DOTATATE PET imaging had markedly superior sensitivity compared to the gold standard of contrast-enhanced MRI (CE-MRI) in identifying primary and recurrent meningioma tissue, irrespective of WHO grade [14]. Because 68Ga-DOTATATE is more sensitive than MRI, it can reveal early or distant tumor spread that conventional imaging may miss.

There is currently no established standard treatment for metastatic meningiomas. Evidence from Phase II trials has shown a modest benefit of systemic therapy. A deeper understanding of meningiomas has been facilitated by genomic and epigenomic investigations, which may be pivotal for improved diagnosis and, importantly, identification of targeted therapies. The novel efforts include mutational analysis and chromosomal instability [15]. DNA methylation-based classifiers provide prognostic stratification beyond histopathology and WHO grade by identifying biologically aggressive meningiomas, including a subset of histologically Grade 1 and Grade 2 tumors associated with early recurrence, aggressive behavior, and inferior survival, and may have offered additional insight into the unexpectedly aggressive and metastatic course observed in this case [16].

Limitations

This report is limited by the scarcity of published literature on metastatic meningiomas, which limits its generalizability. Many older case reports predate modern WHO criteria, making comparisons difficult. In addition, evolving definitions, particularly the inclusion of brain invasion as a Grade 2 criterion, introduce heterogeneity in historical datasets.

## Conclusions

We present a rare case of early pulmonary metastasis from a treated WHO Grade 2 meningioma. This highlights the need to incorporate more diagnostic tools for high-risk patients. Larger studies are needed to better understand risk factors and optimal surveillance strategies for meningiomas with a high potential for metastases.
